# The Impact of Demographic and Clinical Factors on the Quality of Life in Patients with Neurodegenerative Cerebellar Ataxias

**DOI:** 10.3390/brainsci14010001

**Published:** 2023-12-19

**Authors:** Olivera Tamaš, Gorica Marić, Milutin Kostić, Andona Milovanović, Katarina Đurđević, Biljana Salak Đokić, Elka Stefanova, Tatjana Pekmezović, Nataša Dragašević-Mišković

**Affiliations:** 1 Neurology Clinic, University Clinical Centre of Serbia, Faculty of Medicine, University of Belgrade, 11000 Belgrade, Serbia; andona8@gmail.com (A.M.); bsalak@gmail.com (B.S.Đ.); steela21@gmail.com (E.S.); ntdragasevic@gmail.com (N.D.-M.); 2 Institute of Epidemiology, Faculty of Medicine, University of Belgrade, 11000 Belgrade, Serbia; goricamaric87@gmail.com (G.M.); pekmezovic@sezampro.rs (T.P.); 3 Institute of Mental Health, Faculty of Medicine, University of Belgrade, 11000 Belgrade, Serbia; milutin.kostic@imh.org.rs

**Keywords:** neurodegenerative cerebellar ataxias, quality of life, SF-36, impaired mobility, depression

## Abstract

In neurodegenerative cerebellar ataxias, not only ataxia but also extra-cerebellar signs have a significant impact on patients’ health related to quality of life (HRQoL). The aim of this study was to evaluate the various aspects of HRQoL and predictors of QoL in patients with neurodegenerative cerebellar ataxias. We included a total of 107 patients with cerebellar degenerative ataxia. Patients filled out the validated Serbian version of the SF-36 used for the assessment of HRQoL. All patients were clinically evaluated using SARA, INAS, and neuropsychological tests to assess their global cognitive status and different psychiatric scales. The most frequent types of neurodegenerative cerebellar ataxias were autosomal dominant ataxias (38.3%) and sporadic ataxias (32.7%). Mean age at diagnosis was 35.3 ± 16.23 years, and disease duration was on average 12.1 ± 9.91 years. Mean total SF-36 score was 50.63 ± 20.50. Hierarchical regression analysis showed that in the case of the PHC score, the most significant predictors are the patient’s actual age, severity of ataxia, and ACE total score. For MHC, the Hamilton depression score was the most important predictor. Our study has shown that HRQoL measured by SF-36 in patients with neurodegenerative cerebellar disorders is strongly influenced by impaired mobility and depression.

## 1. Introduction

Neurodegenerative cerebellar ataxias represent a complex group of diseases, most of which are genetically determined (hereditary autosomal dominant, autosomal recessive, X-linked ataxias, and mitochondrial ataxias) [1]. 

A group of patients with neurodegenerative ataxias, who have a negative family, is labeled as having sporadic ataxia of unknown cause (SAOA), but recently it was found that a number of them carry new mutations, such as the autosomal recessive biallelic intronic repeat expansion mutation in the form of AAGGG in the *RFC1* gene or the autosomal dominant intronic GAA repeat expansion in the gene that encodes for fibroblast growth factor 14 (*FGF14*) [2,3].

Multiple system atrophy with predominant cerebellar ataxia (MSA-C) symptoms presents with diverse motor, autonomic, and sleep-related symptoms and has a rather severe clinical course that can be difficult to distinguish from SAOA. and other progressive ataxias [4].

Neurodegenerative cerebellar ataxias are characterized by gait and balance abnormalities, appendicular incoordination, abnormalities of eye movement, and speech. In addition to cerebellar, extra-cerebellar involvement, including pyramidal signs, peripheral neuropathy, autonomic dysfunctions, depression, cognitive decline, and psychiatric changes, can also be present [1]. Although both ataxia and extra-cerebellar signs can have a significant impact on patients’ health-related quality of life (HRQoL), a surprisingly small number of studies have examined the HRQoL in degenerative ataxias. Considering the impact of QoL on daily life and functional outcomes, effective interventions should be implemented to improve QoL in these patients.

The aim of this study was to evaluate the various aspects of HRQoL and predictors of QoL in patients with neurodegenerative cerebellar ataxias. 

## 2. Materials and Methods

### 2.1. Study Design

The methodology of this study has been previously described in detail [5]. Briefly, in this retrospective cross-sectional study, we enrolled consecutive neurodegenerative cerebellar ataxias patients, ≥18 years of age, from the Neurology Clinic, University Clinical Center of Serbia, from 2017 to 2020. 

### 2.2. Selection of Participants

We included a total of 107 patients with cerebellar degenerative ataxia, of whom 12 patients had mutations for spinocerebellar ataxia type 1 (SCA1), 7 patients for SCA2, and 1 patient with SCA7, while 21 patients were genetically undetermined but had an apparently autosomal dominant pattern. Out of a total of 21 patients with apparently AR inheritance, 5 had a mutation for Friedreich’s ataxia (FRDA), 7 had an ANO10 mutation, 1 had AR spastic ataxia of Charlevoix-Saguenay (ARSACS), 1 had ataxia with oculomotor apraxia type 2 (AOA2), 3 patients were positive for a mutation in the *RFC1* gene, and 4 patients had an apparently AR inheritance pattern that was genetically undetermined. 

We also included 35 patients with sporadic adult-onset ataxia (SAOA) and 10 patients with multiple system atrophy-cerebellar subtype (MSAc).

Patients with proven symptomatic causes of cerebellar syndrome (metabolic, endocrinological, paraneoplastic, or immunological), previous information on chronic consumption of alcohol, drugs, or toxic substances known to cause ataxia, or fulfilled criteria for establishing a diagnosis of another neurodegenerative disease, as well as patients with any other major psychiatric disorders, were excluded.

The participants’ clinical profiles were recorded, and a detailed clinical examination was conducted. Collected data also included information on age, gender, age at disease onset, and disease duration.

The study was approved by the Ethical Committee of the Faculty of Medicine, University of Belgrade, and all participants signed informed consent. 

### 2.3. Instruments

Several scales were used in this study. While a number of them have been described in the aforementioned study [5], others are detailed here. 

Ataxia severity was assessed using the Scale for Assessment and Rating of Ataxia (SARA) [6], the Spinocerebellar Ataxia Functional Index (SCAFI) [7] and Composite Cerebellar Functional Severity Score (CCFS) [8]. Noncerebellar signs were evaluated using the Inventory of Non-Ataxia Signs (INAS) [9] and autonomic dysfunction using the Autonomic Symptoms Assessment Questionnaire (SCOPA AUT) [10].

### 2.4. Composite Cerebellar Functional Severity Score—CCFS

The patient, who is in a sitting position, holds 9 pins (diameter 9 mm and length 32 mm) in one hand. On the other hand, he places them one by one on a board with nine holes [11]. Timing begins when the patient places the first pin in the hole and ends when the patient places the last one. During the test, the examiner holds the board firmly on the table. Testing is performed with the dominant hand. If the patient lowers the peg, the examiner stops the stopwatch, and the patient does the test from the beginning.

### 2.5. Click Test

It measures the coordination of the index finger. The test is performed by using a simple home device consisting of two mechanical counters placed on a wooden board at a distance of 39 cm. The patient presses the buttons on the counters alternately with their index finger ten times. Timing starts when the patient presses the first button and ends when they press number 10 on the second counter. The test is performed once with the dominant hand.

### 2.6. Spinocerebellar Ataxia Functional Index—SCAFI

We assessed three functional measures: The 8 m walking time (8MW), the 9-hole and peg test (9HPT), and the PATA test for word repetition speed. The 8MW is defined as the time required to walk a distance of 8 m with any aid but without the aid of another person or a wall, “safely but as quickly as possible”. Before calculating the composite functional index scores of each of the subtests (PATA, 9HPT reciprocal, and 8MW reciprocal), they were converted into Z-scores, expressed as the SD of the baseline mean. The Z-score is defined as follows: Z-score (average of the mean value of the study population of each subject in both trials)/SD of the study population. As an average for both trials in the mentioned equation, in the case of 8MW, the reciprocal average for both trials was taken, and in the case of 9HPT, the mean value of the reciprocal average of both hands was taken. In principle, a higher Z-score corresponds to better performance on all three tests.

### 2.7. Inventory of Non-Ataxia Signs—INAS

INAS consists of 30 items, each of which is related to one of the following 16 signs or syndromes: areflexia, hyperreflexia, extensor plantar response, spasticity, paresis, amyotrophy, fasciculations, myoclonus, rigidity, chorea, dystonia, resting tremor, sensory symptoms, brainstem oculomotor signs (horizontal and vertical ophthalmoparesis, slowing of saccades), urinary dysfunction, and cognitive impairment. The INAS score represents a dimensionless value ranging from 0 (absence of signs other than signs of ataxia) to 16 (the most severe case of extra-cerebellar involvement).

### 2.8. Autonomic Symptoms Assessment Questionnaire—SCOPA AUT

To investigate the presence and frequency of autonomic symptoms in our group of patients, we used SCOPA AUT. This refers to 25 questions that are grouped into 6 autonomous domains (gastrointestinal—7), (urinary—6), (cardiovascular—3), (thermoregulation—4), (pupilomotor—1), and sexual (2 questions for men, 2 questions for women). The scale is completed by the patient himself with a ranking of the frequency of occurrence of these symptoms, from 0 (never) to 3 (often). The maximum score on this questionnaire is 69 (a higher score reflects the presence of greater autonomic dysfunction). 

Global cognitive function was evaluated by Addenbrooke’s scale for assessing cognitive function—revised version (ACE-R) [12] and by the Mini-Mental State Examination (MMSE) [13].

### 2.9. Assessment of Anxiety

Anxiety was assessed using the Hamilton Anxiety Rating Scale (HAMA) [14]. The scale is completed by the examiner, a trained physician, based on the interview conducted with the patient. Fourteen symptoms are scored from 0 (not present) to 4 (severe). The total score ranges between 0 and 56, and patients who score ≥13 are considered anxious.

### 2.10. Assessment of Depression

Depression was assessed with the help of the Hamilton Depression Rating Scale (HAMD) [15]. The testing consists of a structured interview carried out by a trained physician. A HAMD score above 17 is considered to be indicative of depression.

### 2.11. Assessment of Apathy

We used the Modified Scale to Assess Apathy (AS, from English Apathy Score) [16], which contains 14 questions. Patients who scored ≥14 were considered apathetic.

### 2.12. 36-Item Short Form Health Survey (SF-36)

Patients filled out the SF-36 (validated Serbian version) [17], a generic questionnaire used for the assessment of HRQoL. SF-36 is used to measure HRQoL in many chronic diseases [18]. Answers for all the items are transformed into values between 0 and 100, and values are aggregated into domain and composite scores. Domains are: Physical functioning (PF), role limitations due to physical health (RP), pain (BP), general health (GH), vitality (VT), social functioning (SF), role limitations due to emotional problems (RE), and emotional wellbeing (EW). SF-36 also provides two summary scores: Physical health composite score (PHC) and mental health composite score (MHC), as well as total score. Scoring and calculation of scales were performed with Ware’s survey manual [19,20]. Answers for all items are transformed into values between 0 and 100, and values are aggregated into domain and composite scores. 

### 2.13. Statistical Analysis

Demographic and clinical characteristics of participants were described using descriptive statistics (mean and standard deviation for continuous parameters and frequencies with percentages for nominal data). SF-36 domain, composite, and total scores were also presented as the mean ± standard deviation. In order to determine independent contributors to the PHC and MHC scores of the SF-36 questionnaire, hierarchical linear regression analyses were used. In these analyses, the dependent variables were PHC or MHC, while in the three models, the same independent variables were included. In the first model, age, sex, and disease duration were entered. In the second model, SARA and INAS total scores were added. Finally, the ACE total score and Hamilton depression, anxiety, and apathy total scores were inserted. A *p*-value less than 0.5 was considered significant. All analyses were performed in SPSS (Statistical Package for Social Sciences), version 20.0.

## 3. Results

The demographic and clinical profiles of participants are shown in Table 1. The study sample (*n* = 107) included 59 (55.1%) males and 48 (44.9%) females, with an average age of 47.5 ± 12.4 years. The majority of the participants were married (59.8%), had one or more children (59.4%), and had on average 11.7 ± 2.5 years of education (Table 1). 

The clinical profile of participants is shown in Table 2. The most frequent types of neurodegenerative cerebellar ataxias were AD (38.3%) and sporadic neurodegenerative cerebellar disorders (32.7%), followed by AR (19.6%) and MSAc (9.3%). The mean age at diagnosis was 35.3 ± 16.23 years, and disease duration was on average 12.1 ± 9.91 years. Mean time to walking support tool was 8.7 ± 6.5 years, and mean time to wheelchair was 12.4 ± 5.9 years. The average SARA total score was 14.9 ± 7.0 years (Table 2). 

Table 3 contains average SF-36 scale scores. Mean total SF-36 score was 50.63 ± 20.50. Mean PHC was 43.60 ± 20.33, and mean MHC was 57.67 ± 23.77. When the domain scores were analyzed, the highest scale scores were obtained for the pain domain (70.80 ± 28.31), while the lowest scores were reported for general health (34.10 ± 14.47), physical functioning (34.11 ± 27.97), and role limitation due to physical health (35.38 ± 40.22).

Correlations of different demographic and clinical parameters with PHC and MHC SF-36 scores are presented in Table 4. PHC and MHC significantly correlated with all clinical characteristics of participants.

Table 5 and Table 6 present the results of hierarchical linear regression analyses. In Table 5, it can be seen that, when PHC was set as a dependent variable, all independent variables together explained a total of 39% of the variance in the PHC score (Model 3). More precisely, the first model, including age, sex, and disease duration, explained 4% of the variance (*p* > 0.05). Adding SARA and INAS total scores (Model 2) explained an additional 28% of the variance (*p* < 0.01). Finally, the inclusion of ACE total and Hamilton depression, anxiety, and apathy scores in the third model increased the percentage of variance explained by 7% (*p* < 0.01) (Table 5). Similarly, in the second hierarchical regression analysis, when the dependent variable was MHC, a total of 43% of the variance in this score was explained (Model 3). In Model 1 presented in Table 6 (same independent variables in both hierarchical regression analyses), the percentage of the variance explained was 6% (*p* > 0.05). Variables in the second model explained an additional 17% (*p* < 0.01), and finally, in the third model, the percentage of variance explained increased by 20% (*p* < 0.01).

## 4. Discussion

The main finding of our study is that HRQoL is significantly reduced in the neurodegenerative cerebellar ataxias population, and this is particularly evident in the physical dimensions of QoL, while the mental dimensions were affected to a comparatively lesser degree. Only a few studies have evaluated the HRQoL of people with ataxia, and this study is the first to examine HRQoL among neurodegenerative cerebellar ataxia patients in Serbia. 

Domain score analyses showed that the highest scale scores were obtained for the pain domain, as confirmed by another study [20]. In a large study with 526 patients from Europe, it was reported that SCA3 patients had more frequent pain symptoms than patients with SCA1, SCA2, and SCA6 [21]. The lowest scores were reported for general health, physical functioning, and role limitation due to physical health, similar to another study [22]. Our results are very similar to the main conclusion of the study group that first investigated and identified factors that may be relevant to QoL for people living with ataxia—that impaired mobility was associated with reduced HRQoL [23]. While the validity of this work remains questionable and difficult to interpret, the study represented an early attempt to involve people with ataxia in the research. 

Hierarchical regression analysis showed that in the case of the PHC score, the most significant predictors are the patient’s actual age, severity of ataxia, and ACE total score, i.e., a higher PHC score was registered in younger patients with less severe disease and a higher ACE score. In our study, the actual age was 47.5, which represents middle age. As people of this age are normally working people who lead relatively active lives, the restrictions brought upon by the disease can significantly affect their QoL. One of the hypotheses described earlier is that adaptation to disease-related change may be more difficult after career, relationships, and education become established parts of life [24,25]. In contrast, studies in patients with FRDA indicated that a higher age at the time of examination and a later onset of the disease were associated with fewer limitations and a better perception of health, probably due to the fact that the perception of the youngest patients about their limitations in physical activity deviated more from their age reference group than that of the oldest patients [26].

It was expected that disease severity would have a negative association with the physical aspects of QoL. However, studies that suggest that the burden of the neurological disease process started to impair HRQoL well before the onset of gait ataxia [25,27] caution against making simplistic inferences about the effect of severity and stage of disease as well as age of onset on QoL. Moreover, disease duration in our patients did not have a significant impact on HRQOL, which is in line with previous observations. This means that patients might perceive a decrease in the quality of life in stages when ataxia is not so pronounced [28].

Although we could expect that the presence of neuropathy, pyramidal weakness, dysphagia, and other non-cerebellar signs contributes to the physical and mental aspects of QoL, we showed that the INAS score is not a significant predictor as opposed to the SARA scale. 

In our study, a higher ACE score was a parameter for global cognitive functioning. Since the cerebellum plays a critical role in various cognitive and behavioral controls [29,30,31,32,33] is intuitive that a higher ACE total score leads to better HRQoL. 

In some studies, the severity of ataxia in patients with neurodegenerative cerebellar disorders is positively correlated with the patients’ cognitive deficit [34], although some sources suggest that cognitive functions are affected first and have a slower progression than motor dysfunctions [35]. However, scientists still disagree as to the exact role of the cerebellum in cognition [36]. Patients with cerebellar disturbances have significant and relevant deficits in the working memory, visuospatial, language, and executive function (EF) domains [37,38]. Our findings corroborate evidence in the literature regarding the effect of cognitive functioning on quality of life in other neurodegenerative disorders. Similarly to findings in Parkinson’s disease, worse performance on cognitive assessments was associated with worse quality of life [39].

For MHC, in hierarchical regression analysis, the Hamilton depression score was the most important predictor, with higher scores corresponding to lower MHC. Depression and depressive symptoms were the most common psychiatric manifestations in several studies of patients with cerebellar disorders [40,41]. Depression and other symptoms such as insomnia, fatigue, and anxiety are commonly known to be pervasive among patients with neurodegenerative cerebellar disorders [42]. Depression emerged as a significant predictor of QoL amongst different types of SCA [43]. The potential origins of depression are manifold, and there are ever-present concerns about how depression may impact disease progression. Moreover, it is well known that depression may be a potential confounding factor in the self-evaluation of HRQoL, in which depressed patients overestimate their difficulties. Finally, [25] established that while mental health improved with disease duration, late onset was associated with poorer QoL. This suggests that participants may have experienced difficulty adapting to long-term progressive disease after having lived a long life without disease.

The three previously published papers [20,22,25] used generic instruments to measure HRQoL for people living with ataxia—the Medical Outcomes Study 36-Item Short Form Health Survey or the Euro-qual 5D, also used in our study. Another similarity with our study was that they all explored QoL for progressive ataxia (sporadic progressive ataxia, FRA, or SCA). Taken together, the findings from these studies indicated that people living with progressive ataxia experienced reduced QoL compared to healthy controls in motor domains such as physical functioning as well as non-motor domains such as social functioning, role limitation, and general health perception.

The importance of our study lies in the fact that we evaluated the presence not only of cerebellar but also of noncerebellar features that may be related to the QoL of neurodegenerative cerebellar ataxia patients and not limited to depression, which is what other studies have mostly done. Our study included patients with the predominance of AD and other neurodegenerative cerebellar ataxias who were diagnosed at an average age of 35 years, and at the moment of investigation, the mean disease duration was about 12 years. However, despite the small number of included participants and heterogeneity with regards to the type of ataxia, age at onset, mode of genetic transmission, and clinical features, our data may be valuable because neurodegenerative cerebellar ataxias are rare disorders. 

It is important to address several limitations of our study. One of them is the heterogeneity of groups as well as the lack of standards for the SF-36 questionnaire in the Serbian population, due to which it is impossible to compare the HRQoL of patients with neurodegenerative cerebellar disorders with that of the standard populations. Additionally, generic HRQoL questionnaires such as SF-36 frequently do not adequately illustrate key factors important for certain diseases and events that are related to health [44].

## 5. Conclusions

Neurodegenerative forms of cerebellar ataxias present a progressive and inevitable worsening of the clinical picture, leading to the patients’ functional disability and a serious limitation of autonomy and QoL. Our study has shown that HRQoL measured by SF-36 in patients with neurodegenerative cerebellar disorders is strongly influenced by impaired mobility and depression. This study, in conjunction with other supporting data, has the potential to provide direction for further actions. Similar concerns prompted the call for the development of condition-specific and patient-based measures capable of providing further knowledge and understanding about the impact of ataxia on QoL.

## Figures and Tables

**Table 1:** Demographic profile of participants.

Variable	Value	Range
Sex (%)		
Male	59 (55.1)	
Female	48 (44.9)	
Age (years)	47.5 ± 12.4	(19–69)
BMI (kg/m^2^)	23.5 ± 3.2	(16.4–34.6)
Marital status (%)		
Not married	30 (28.0)	
Married	64 (59.8)	
Divorced	11 (10.3)	
Widowed	2 (1.9)	
Children (%)		
None	43 (40.6)	
One	15 (14.2)	
Two	42 (39.6)	
Three	6 (5.7)	
Handedness (%)		
Right-handed	103 (96.3)	
Left-handed	4 (3.7)	
Education (years)	11.7 ± 2.5	(4–20)
Employed (%)	33 (30.8%)	
Unemployed	56 (52.3%)	
Retired	18 (16.8%)	

**Table 2:** Clinical profile of participants.

Variable	Value	Range
Age at onset (years)	35.3 ± 16.2	(0–68)
Disease duration (years)	12.1 ± 9.9	(1–43)
Time to walking support tool (years)	8.7 ± 6.5	(0.3–25)
Time to wheelchair (years)	12.4 ± 5.9	(1–25)
SARA total score	14.9 ± 7.0	(2–40)
CCFS total score	1.0 ± 0.2	(0–1.78)
SCAFI total score	0.0 ± 0.8	(−2.1–2.4)
INAS total score	4.5 ± 1.9	(1–9)
SCOPA AUT—total autonomic score	4.7 ± 6.5	(0–34)
ACE-R total score	79.1 ± 15.8	(35–100)
MMSE	25.1 ± 4.9	(9–30)
HAMD	8.3 ± 6.0	(0–23)
HAMA	8.3 ± 6.8	(0–25)
Hamilton apathy	10.8 ± 8.3	(0–38)
Disease (%)		
AD	41 (38.3)	
AR	21 (19.6)	
Sporadic	35 (32.7)	
MSAc	10 (9.3)	

Scale for Assessment and Rating of Ataxia (SARA), Spinocerebellar Ataxia Functional Index (SCAFI), Composite Cerebellar Functional Severity Score (CCFS), Inventory of Non-Ataxia Signs (INAS), Autonomic Symptoms Assessment Questionnaire (SCOPA AUT) Mini Mental State Examination (MMSE), Addenbrooke’s Cognitive Examination—Revised Version (ACE-R), Hamilton Depression Rating Scale (HAMD) Hamilton Anxiety Rating Scale (HAMA), autosomal dominant (AD), autosomal recesive (AR), multiple system atrophy-cerebellar subtype (MSAc).

**Table 3:** SF-36 domain, composite, and total score.

SF-36 Domain	Total
Physical functioning	34.11 ± 27.97
Role limitations due to physical health	35.38 ± 40.22
Role limitations due to emotional problems	57.58 ± 44.71
Energy	50.24 ± 23.60
Emotional wellbeing	63.43 ± 20.70
Social functioning	59.43 ± 23.07
Pain	70.80 ± 28.31
General health	34.10 ± 14.47
Physical health composite	43.60 ± 20.33
Mental health composite	57.67 ± 23.77
Total	50.63 ± 20.50

36-Item Short Form Health Survey.

**Table 4:** Correlations between SF-36 composite scores and patients’ demographic and clinical characteristics.

	Age	Sex	Disease Duration	SARA Total	INAS Total	ACE-R Total	HAMD	HAMA	Hamilton Apathy
PHC	−0.127	−0.038	**−0.190** *	**−0.536** **	**−0.331** **	**0.440** **	**−0.406** **	**−0.269** **	**−0.345** **
MHC	−0.144	0.026	−0.108	**−0.388** **	**−0.203** *	**0.318** **	**−0.579** **	**−0.462** **	**−0.493** **

Physcial health composite (PHC), Mental health composite (MHC), Scale for Assessment and Rating of Ataxia (SARA), Inventory of Non-Ataxia Signs (INAS), Addenbrooke’s Cognitive Examination—Revised version (ACE-R), Hamilton Depression Rating Scale (HAMD), Hamilton Anxiety Rating Scale (HAMA). Bold values denote statistical significance, * *p* < 0.05; ** *p* < 0.01.

**Table 5:** Predictors of SF-36 physical health composite score.

Variable	Model 1			Model 2			Model 3		
	B	SE (B)	β	B	SE (B)	β	B	SE (B)	β
Age	−0.21	0.16	−0.13	−0.41	0.15	**−0.25** **	−0.37	0.15	**−0.23** *
Sex	−1.76	4.04	−0.04	−2.87	3.46	−0.07	−2.26	3.40	−0.06
Disease duration	−0.35	0.20	−0.17	0.08	0.18	0.04	0.16	0.19	0.08
SARA total				−1.53	0.32	**−0.49** **	−1.15	0.34	**−0.37** **
INAS total				−1.40	1.08	−0.13	−1.02	1.06	−0.10
ACE-R total							0.31	0.15	**0.21** *
HAMD							−1.10	0.62	−0.32
HAMA							0.36	0.45	0.12
Hamilton apathy							0.22	0.34	0.09
*R* ^2^	0.04			0.32			0.39		
*F* for change in *R*^2^	1.660			**9.415** **			**6.774** **		

B—unstandardized beta coefficient; SE (B)—standard error for beta; β—standardized beta; Scale for Assessment and Rating of Ataxia (SARA), Inventory of Non-Ataxia Signs (INAS), Addenbrooke’s Cognitive Examination—Revised version (ACE-R), Hamilton Depression Rating Scale (HAMD), Hamilton Anxiety Rating Scale (HAMA). Bold values denote statistical significance, * *p* < 0.05; ** *p* < 0.01.

**Table 6:** Predictors of SF-36 mental health composite score.

Variable	Model 1			Model 2			Model 3		
	B	SE (B)	β	B	SE (B)	β	B	SE (B)	β
Age	−0.31	0.19	−0.16	−0.48	0.18	**−0.25** **	−0.18	0.17	−0.09
Sex	1.27	4.70	0.03	0.12	4.31	0.00	−0.48	3.83	−0.01
Disease duration	−0.41	0.23	−0.17	−0.02	0.23	−0.01	−0.06	0.21	−0.03
SARA total				−1.51	0.40	**−0.42** **	−0.67	0.39	−0.19
INAS total				−0.72	1.35	−0.06	−0.25	1.19	−0.02
ACE-R total							0.06	0.17	0.03
HAMD							−1.96	0.70	**−0.49** **
HAMA							0.41	0.50	0.12
Hamilton apathy							−0.38	0.38	−0.13
*R* ^2^	0.06			0.23			0.43		
*F* for change in *R*^2^	2.015			**5.812** **			**8.059** **		

B—unstandardized beta coefficient; SE (B)—standard error for beta; β—standardized beta; Scale for Assessment and Rating of Ataxia (SARA), Inventory of Non-Ataxia Signs (INAS), Addenbrooke’s Cognitive Examination—Revised version (ACE-R), Hamilton Depression Rating Scale (HAMD), Hamilton Anxiety Rating Scale (HAMA). Bold values denote statistical significance, ** *p* < 0.01.

## Data Availability

The data from this study are available on request from the corresponding author. The data are not publicly available due to privacy and ethical restrictions.
